# The PPARα-PGC-1α Axis Controls Cardiac Energy Metabolism in Healthy and Diseased Myocardium

**DOI:** 10.1155/2008/253817

**Published:** 2007-11-25

**Authors:** Jennifer G. Duncan, Brian N. Finck

**Affiliations:** Center for Cardiovascular Research, Departments of Pediatrics and Medicine, Washington University School of Medicine, 660 S. Euclid Avenue Campus Box 8031, Saint Louis, MO 63110, USA

## Abstract

The mammalian myocardium is an omnivorous organ that relies on multiple substrates in order to fulfill its tremendous energy demands. Cardiac energy metabolism preference is regulated at several critical points, including at the level of gene transcription. Emerging evidence indicates that the nuclear receptor PPARα and its cardiac-enriched coactivator protein, PGC-1α, play important roles in the transcriptional control of myocardial energy metabolism. The PPARα-PGC-1α complex controls the expression of genes encoding enzymes involved in cardiac fatty acid and glucose metabolism as well as mitochondrial biogenesis. Also, evidence has emerged that the activity of the PPARα-PGC-1α complex is perturbed in several pathophysiologic conditions and that altered activity of this pathway may play a role in cardiomyopathic remodeling. In this review, we detail the current understanding of the effects of the PPARα-PGC-1α axis in regulating mitochondrial energy metabolism and cardiac function in response to physiologic and pathophysiologic stimuli.

## 1. INTRODUCTION

The myocardium has an enormous and steady demand for energy that is met through high-level mitochondrial oxidative metabolism. Glucose, lactate, and fatty acids are all oxidized in the mitochondrion to produce reducing equivalents required for ATP synthesis in the process of oxidative phosphorylation (OXPHOS). Much of the mitochondrial-derived ATP is then transported to the cytoplasm, making energy available for cellular work, which includes its crucial role in cardiac myocyte contraction. Acute changes in flux through these metabolic pathways are mediated by changes in substrate concentrations and covalent or allosteric modification of enzymes catalyzing these reactions. However, the capacity for mitochondrial oxidative metabolism is also mediated at the level of gene transcription [1].

Work in several labs has demonstrated that the three PPAR isoforms (PPARα, β/δ, and γ) are expressed, to varying degrees, in the myocardium and play important roles in the transcriptional regulation of cardiac metabolism and function. The ability to modulate PPAR activity with specific activating ligands as well as genetic activation or deactivation in mice has enriched our understanding of the importance of each of the various PPAR isoforms in determining cardiac metabolism, structure, and function. However, given the limited space available in this review, we will focus our attention on the PPARα isoform and its coactivator protein PGC-1α.

## 2. PPARα AND MYOCARDIAL FATTY ACID METABOLISM

The PPARα isoform is robustly expressed in the parenchymal cells of the adult heart and plays an important role in regulating cardiac myocyte metabolism [2, 3]. In the myocardium, PPARα activation induces the expression of genes encoding nearly every step in the cellular fatty acid utilization pathway including (i) fatty acid transport proteins that facilitate fatty acid entry into the cell, (ii) acyl-CoA synthetases that esterify fatty acids to coenzyme A and prevent their efflux, (iii) fatty acid binding proteins that shuttle fatty acids to various cellular compartments, (iv) proteins that catalyze the import of fatty acids into the mitochondrion, (v) every enzyme in the mitochondrial fatty acid β-oxidation spiral, and (vi) various accessory components of fatty acid metabolism (e.g., uncoupling proteins).

Administration of PPARα ligand to rodent models results in a robust activation of PPAR target genes in liver, but the effects of in vivo ligand administration on cardiac gene expression is minimal [4]. Indeed, PPARα agonist administration to diabetic mice actually leads to diminished cardiac fatty acid utilization [5, 6], possibly by reducing the exposure of the heart to triglyceride-rich lipoproteins or endogenous fatty acid ligands. It is unclear whether PPARα ligand administration targets the heart directly in humans; and there are likely differences in the PPAR response between the species. Due to the hepatic specific effects of PPARα ligands in rodents, much of our knowledge regarding the target pathways of PPARα in myocardium is based on studies with genetic alterations in PPARα activity. Mice with constitutive deletion (in all tissues) of the gene encoding PPARα (PPARα null mice) exhibit diminished rates of cardiac fatty acid oxidation (FAO) and increased reliance on glucose utilization pathways [7–9]. This shift is mediated, at least in part, by diminished expression of several genes involved in FAO [10] and a concomitant increase in the expression of genes encoding proteins involved in glucose uptake and utilization [7]. At the other end of the metabolic spectrum, we have characterized transgenic mice overexpressing PPARα in a cardiac-restricted manner (MHC-PPARα mice) [8, 11–16]. The expression of many genes involved in fatty acid uptake and utilization is upregulated in MHC-PPARα mice, while the expression of glucose transporter and glycolytic enzymes is strikingly suppressed [11]. Consistent with this pattern of metabolic gene expression, MHC-PPARα mice rely almost exclusively on FAO and use very little glucose [8, 9, 11]. In summary, the opposing metabolic phenotypes of these transgenic models with activation or deactivation of PPARα support an important role for PPARα in regulating cardiac energy metabolism.

## 3. THE PGC-1α TRANSCRIPTIONAL COACTIVATOR AND THE CONTROL OF CARDIAC ENERGY METABOLISM

**Figure 1:**
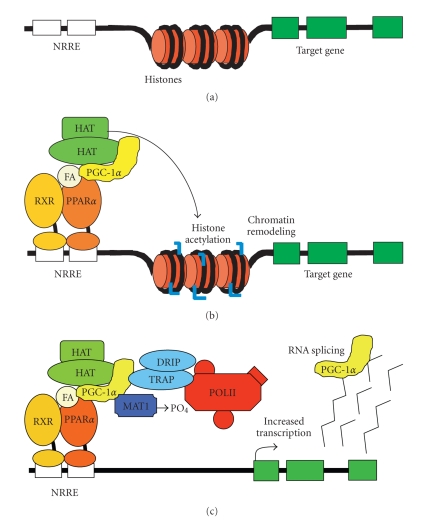
*Mechanisms of PPARα activation and PGC-1α coactivator activity.* Depiction of a potential PPARα target gene and nuclear receptor response element (NRRE) within the promoter region in the nonactivated state *(top)*. PPARα activation by fatty acid (FA) ligand leads to binding to the NRRE with its heterodimeric partner RXRα; and its coactivator PGC-1α. PGC-1α recruits additional coactivators with histone acetyltransferase (HAT) activity, which promotes chromatin unwinding and increases RNA polymerase II (POL II) access to the target gene promoter *(middle)*. PGC-1α also interacts with the TRAP/DRIP complex and with ménage-à-trois 1 (MAT1) which phosphorylates POL II to increase the probability of gene transcription. In addition, PGC-1α plays a role in RNA splicing via an RNA processing domain in its C-terminus *(bottom)*.

 Transcriptional coactivators are a group of proteins that control gene expression via protein-protein interactions with DNA-bound transcription factors, including PPARα (Figure 1). Although several transcriptional coactivators are known to interact with PPARα, in the heart, the physical and functional interaction with PPARγ coactivator 1α (PGC-1α) has been best described. PGC-1α was originally discovered in a yeast two-hybrid screen for proteins that interacted with the PPARγ isoform and that were enriched in a brown adipocyte library [17]. Based on sequence homology in some highly conserved regions, two additional PGC-1 family members have now been identified (PGC-1β and PGC-related coactivator (PRC)) [18, 19].

Coactivators are broadly categorized into two classes. Class I coactivators regulate gene transcription through enzymatic modification of chromatin (e.g., acetylation and methylation), which facilitates DNA unwinding and enhances the probability that a gene will be transcribed by the RNA polymerase II complex. Class II coactivators work by interacting with the RNA polymerase machinery (e.g., RNA polymerase II or the TRAP/DRIP complex) [20, 21]. PGC-1α functions as a Class II coactivator since it does not possess intrinsic chromatin modifying activity and interacts directly with the TRAP/DRIP complex to link with RNA polymerase II (Figure 1) [20]. PGC-1α also recruits Class I coactivators with histone acetyltransferase activity to chromatin in the target gene promoter [20, 22] and docks with a protein called ménage-à-trois 1, which phosphorylates RNA polymerase II to modulate its activity (Figure 1) [23]. Finally, PGC-1α possesses an RNA processing domain that may also contribute to its transcriptional regulatory function [24].

PGC-1 interacts with and coactivates a broad array of transcription factors to transduce developmental, nutritional, and physiological stimuli to the control of diverse cellular energy metabolic pathways [25, 26]. In heart, PGC-1α has thus far been linked with 3 families of transcription factors: (i) the PPAR family, (ii) the estrogen-related receptor (ERR) family, and (iii) nuclear respiratory factor 1 (NRF-1). The interaction between PGC-1α and PPARα serves to control the expression of enzymes involved in fatty acid uptake and oxidation [27] and possibly proteins involved in the process of mitochondrial biogenesis [15]. The ERR family (ERRα, β, γ) of orphan nuclear receptors is also an important cardiac PGC-1α target that drives increased expression of genes encoding FAO and OXPHOS enzymes [28–31]. Finally, NRF-1 is a nuclear-encoded transcription factor that is coactivated by PGC-1α to regulate transcription of genes involved in mitochondrial OXPHOS, mtDNA transcription and replication, and mitochondrial biogenesis [32–35]. Additional details regarding PGC-1-mediated control of energy metabolism through ERRα and NRF-1 can be found in other recent reviews [26, 35–37].

Several genetically-engineered mouse models have been used to probe the role of PGC-1α in regulating cardiac metabolism. Mice that constitutively overexpress PGC-1α in the myocardium exhibit profound mitochondrial proliferation, cardiomyopathy, and early death secondary to heart failure [33]. The severity of the cardiomyopathy in this model precluded a full investigation of the pathologic mechanisms that contribute to cardiac dysfunction. To address this issue, a second model evaluated overexpression of PGC-1α in the heart using a tetracycline-inducible system [38]. This model revealed dramatic mitochondrial proliferation when PGC-1α was overexpressed in the neonatal phase, without overt effects on cardiac function. In contrast, overexpression of PGC-1α in adult mice provoked only modest mitochondrial proliferation, but led to abnormal mitochondrial and myofibril architecture and severe cardiac dysfunction [38]. Interestingly, cardiomyopathy in these mice was completely reversible by discontinuing PGC-1α overexpression [38]. These gain-of-function strategies indicate that PGC-1α plays important roles in regulating multiple aspects of myocardial metabolism and is a strong stimulus for the process of mitochondrial biogenesis.

The cardiac phenotype of two separate lines of mice with constitutive PGC-1α deficiency also support an important role for PGC-1α in cardiac metabolism and function [39–41]. Both lines of PGC-1α-deficient mice exhibit impaired mitochondrial OXPHOS function and decreased expression of many genes encoding enzymes in mitochondrial metabolic pathways. PGC-1α deficiency also leads to cardiac dysfunction, especially in the context of pathophysiologic stimuli like pressure overload-induced cardiac hypertrophy [40, 41]. Interestingly, the severity of the cardiac functional phenotype varies between the two lines of knockout mice. One line exhibits age-associated cardiac dysfunction that is manifested by 7-8 months old as left ventricular chamber dilatation, diminished fractional shortening, and an activation of gene markers of cardiomyopathy [41]. Conversely, the other line of knockout mice exhibits no signs of cardiac dysfunction, but displays diminished chronotropic capacity in response to a β-adrenergic stimulus [39]. The mechanistic basis for this disparity in the two mouse mouse models is unknown. Collectively, these gain- and loss-of-function studies demonstrate that PGC-1α has a critical role in control of cardiac energy metabolism.

## 4. PPARα-PGC-1α-MEDIATED CONTROL OF METABOLISM IN RESPONSE TO DEVELOPMENTAL OR PHYSIOLOGIC CUES

**Figure 2:**
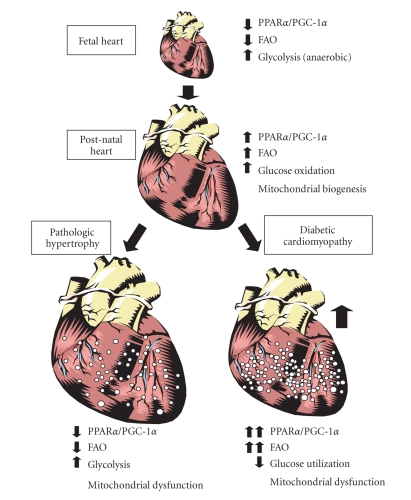
*Dynamic regulation of PPARα-PGC-1α complex activity in developing, failing, and diabetic heart.* Physiological cardiac growth resulting from postnatal maturation is associated with increased PPARα and PGC-1α expression and marked expansion of mitochondrial volume density and oxidative capacity. Conversely, pathologic hypertrophy is linked to decreased PPARα-PGC-1α expression and/or activity and diminished reliance on oxidative mitochondrial metabolism often leading to intramyocellular lipid accumulation. Finally, in the diabetic heart, PPARα-PGC-1α complex activity is increased along with the cardiac reliance on FAO. Despite of high-level FAO, the cardiac lipid accumulation is a hallmark of the diabetic heart and lipotoxicity may play a key role in the development of diabetic cardiomyopathy.

Myocardial energy substrate preference is remarkably pliant and the heart can rapidly modulate fuel utilization depending upon the developmental stage, nutritional context, or disease state [42]. The PPARα-PGC-1α complex plays an important role in catalyzing these changes. For example, the fetal heart utilizes predominantly anaerobic glucose metabolism to fulfill its energy needs. However, almost immediately after birth, a rapid and profound developmental shift occurs. The workload of the heart is increased and the availability of fatty acids and oxygen for fuel becomes much greater (Figure 2). In response to these changes, the myocardium increases its reliance on mitochondrially derived ATP as an energy source through a coordinated induction of mitochondrially and nuclear-encoded genes involved in mitochondrial metabolism, structure, and function [43–45]. This developmental shift is accompanied by a robust activation of the PPARα-PGC-1α system [33, 43]; and it is likely that these two factors play a crucial role in this developmental switch.

Fasting is another physiologic context associated with a marked increase in PPARα-PGC-1α activity. To “spare” glucose for other organs that lack the capacity to catabolize fatty acids, the heart markedly increases its use of fatty acids under conditions of food deprivation [42]. Although the expression of the gene encoding PPARα is unaltered, the expression of PGC-1α is strongly induced [33]. Together with heightened availability of fatty acids that act as endogenous ligands for PPARα, this serves to rapidly amplify PPARα transcriptional activity. In fact, the expression of the broad program of myocardial FAO enzymes is markedly induced by food deprivation and this response is significantly blunted in mice lacking PPARα [10]. In sum, the PPARα-PGC-1α complex serves to regulate the capacity for FAO in response to physiologic cues that signal an increased need for mitochondrial fatty acid utilization.

## 5. ALTERED PPARα-PGC-1α SIGNALING IN THE FAILING HEART

Cardiac energy substrate metabolism is perturbed in the hypertrophied and failing heart, reverting to a program of energy substrate metabolism similar to the “fetal” profile (Figure 2). Specifically, the myocardium shifts from dependence on FAO towards glucose utilization; primarily anaerobic glycolysis [46–49]. Importantly, this switch in energy substrate preference detected in various experimental models is also observed in humans with idiopathic dilated cardiomyopathy [50]. These changes in energy substrate preference are mediated, at least in part, by a downregulation of the genes encoding enzymes involved in FAO, OXPHOS, and the PPARα-PGC-1α complex [3, 48, 51–60]. The expression of the genes encoding PPARα and PGC-1α is known to be diminished in several rodent models of pressure overload or hypertensive heart disease [3, 40, 61], pacing-induced heart failure [62, 63], hypoxia [52], ischemic heart disease [55, 58, 59, 64], as well as genetically engineered models of heart failure [65–67]. The molecular mechanisms whereby pathologic stimuli lead to a transcriptional downregulation of PPARα and PGC-1α are not well understood, but may involve reactive oxygen species generation [64]. In addition, under pathologic conditions, PPARα activity is inhibited post-translationally through lower levels of the obligate heterodimeric partner of PPARα, retinoid X receptor (RXR) [57], and direct phosphorylation by the extracellular signal-related kinase and mitogen-activated protein kinase (ERK-MAPK) pathway [3]. These findings suggest that deactivation of the cardiac PPARα-PGC-1α axis in failing heart is a key component of the observed shift in energy metabolism. In support of this, reactivation of PPARα or PGC-1α prevents the downregulation of oxidative gene expression that occurs in cardiac myocytes challenged with pathologic stimuli [61, 63–65, 68, 69]. Experimental models have found altered metabolism and gene expression in both the hypertrophied and the overtly failing heart, but longitudinal evaluation of progressive changes in the PPARα-PGC-1α axis has not been done. Studies to evaluate the sequence of events will be crucial to understanding the role of altered metabolic regulation in disease progression.

One point that remains to be addressed is whether deactivation of oxidative metabolism and the PPARα-PGC-1α complex in the hypertrophied and failing heart is adaptive or maladaptive. The shift towards glycolysis allows continued ATP production with less oxygen consumption, and thus would appear to be an adaptive response. Indeed, overexpression of the GLUT1 glucose transporter prevented cardiac dysfunction in response to pressure overload [70]. Partial inhibitors of FAO also produce positive inotropic effects in patients with ischemic and nonischemic heart disease [71–76]. Ligand-mediated activation of PPARα in models of pressure overload [61] or ischemia [64] exacerbated ventricular dysfunction and pathologic remodeling. However, other reports show no ill effects of PPARα agonism or increased FAO in pathologic conditions [68, 69, 77]. Moreover, there is abundant evidence that chronic shifts towards glycolysis are maladaptive. Most reports suggest that PPARα agonists are beneficial in the response to ischemia [78–80] and various models of heart failure [63, 81–83]. Similarly, PGC-1α overexpression rescued the cardiac myocyte dysfunction and apoptosis in a mouse model of cardiomyopathy [65]. Mice with chronic reliance on glucose metabolism due to loss of cardiac lipoprotein lipase develop cardiac dysfunction with age and demonstrate significant mortality associated with the stress of aortic banding [84]. Finally, PPARα deficient animals that shift metabolism predominantly towards glucose oxidation exhibit age-associated cardiac fibrosis [85] and were unable to respond to increased workload and developed energy depletion [86].

The concept that the myocardium must maintain metabolic flexibility and a balance of substrate utilization during pathologic remodeling has recently pushed to the forefront. However, the biologic basis for this concept is unclear. It may be that chronic reliance on glucose as the predominant substrate is insufficient for ATP production in failing heart. Compared to FAO, glycolysis produces much less ATP per mole of substrate and there is evidence that long-term reliance on glycolysis leads to ATP deficiency in failing heart. Indeed, the phospho-creatine/ATP ratio is reduced in failing heart [49, 87–89] and a decline in this ratio is predictive of impending mortality in human heart failure patients [90]. The idea that energy starvation plays a significant role in the development of heart failure is also supported by severe cardiomyopathies in animal models with deletions in FAO enzymes [91, 92] or enzymes involved in mitochondrial ATP production [93–95]. Moreover, humans with inborn errors in these pathways often present with cardiomyopathy [96]. It is also possible that impairments in rates of FAO in failing heart are maladaptive because they lead to myocardial lipid accumulation (lipotoxicity) [97], which is linked to cardiac dysfunction [98–100]. Alternatively, or in addition, the inability to switch energy substrate preference in the context of changes in substrate availability could also contribute to pathologic remodeling.

## 6. PPARα AND PGC-1α IN THE DIABETIC HEART

Cardiovascular disease is exceptionally prevalent in patients with diabetes. Although the prevalence of dyslipidemias and hypertension certainly contributes to cardiovascular risk in diabetic subjects, cardiomyopathy is highly prevalent independent of these risk factors. Cardiomyopathy in diabetic subjects that occurs in the absence of known risk factors is often termed “diabetic cardiomyopathy” [101–104]. Unfortunately, the etiology of diabetic cardiomyopathy is poorly understood.

Evidence has emerged that abnormalities in myocardial energy metabolism play a significant role in the pathogenesis of diabetic cardiomyopathy. Indeed, in experimental models of uncontrolled diabetes (type 1 or 2), cardiac energy substrate flexibility becomes constrained and the diabetic heart relies almost exclusively on mitochondrial FAO for its ATP requirements [105–108]. Recently, these metabolic observations from animal models have also been confirmed in human subjects with type 1 diabetes [109]. The expression of PPARα, PGC-1α, and many target genes involved in FAO are increased in the murine insulin-resistant [15] and diabetic heart (type 1 and type 2) [11, 110, 111] and may play a key role in the observed metabolic switch to FAO. PPARα deficiency in the setting of insulin resistance [15] or diabetes [110] blunts activation of FAO gene expression, suggesting that activation of the PPARα-PGC-1α regulatory network is critical for the increased FAO rates and lipid uptake seen in the diabetic heart. Consistent with this, transgenic mice that overexpress PPARα exclusively in the heart (MHC-PPARα mice) have a cardiac metabolic phenotype similar to that observed in diabetic heart, including accelerated rates of FAO, a striking diminution in glucose uptake and utilization, and a mitochondrial biogenic response [11, 15]. We have also observed that high-level fatty acid utilization in hearts of MHC-PPARα mice leads to the development of cardiac hypertrophy and dysfunction [11, 12]. We believe that sustained activation of the PPARα-PGC-1α complex in the insulin-resistant and diabetic heart promotes a state of metabolic inflexibility that leads to cardiomyopathic remodeling.

Despite high rates of FAO, myocardial lipid accumulation is a hallmark of the diabetic heart [112–116]. Prolonged accumulation of fats in the myocardium is believed to be highly toxic and is linked to the development of insulin resistance and cardiac dysfunction [12, 98–100, 114]. Our data suggest that PPARα drives this lipotoxic response in diabetic heart. The cardiomyopathic phenotype is relatively mild in unchallenged MHC-PPARα mice, but when the transgenic mice were given a high-fat diet, the cardiomyopathic phenotype was strikingly exacerbated; and mice exhibited clinical signs of heart failure, including depressed fractional shortening and ventricular chamber dilatation [12]. Pathologic remodeling in MHC-PPARα mice was accompanied by marked cardiac lipid accumulation. Moreover, genetic ablation of the fatty acid transporter CD36 in the context of PPARα overexpression prevents high-fat diet-induced cardiac lipid accumulation and dysfunction [16]. Finally, ligand-mediated activation of PPARα also drives lipid accumulation and an adverse outcome following ischemic insult [64]. These findings suggest that PPARα-driven lipotoxicity could be an important mechanism in cardiomyopathic remodeling of the diabetic heart.

Other components of the metabolic derangements in diabetic heart are abnormalities in mitochondrial ultrastructure and function [15, 111, 117–120]. Mitochondria isolated from diabetic rodents exhibit depressed rates of OXPHOS [117, 118] and diminished efficiency in ATP synthesis [120, 121], likely due to increased uncoupled respiration [121]. Mitochondrial proliferation is common in hearts of diabetic rodents [15, 119, 121, 122]. However, mitochondria from both type 1 and type 2 diabetic hearts often exhibit ultrastructural abnormalities, including degenerative cristae [15, 119]. The literature regarding the effects of insulin resistance and diabetes on mitochondrial gene expression is mixed with some reports showing an activation [15, 119] and others showing deactivation [123, 124]. We recently found that mitochondrial biogenesis and OXPHOS gene expression are increased in a mouse model of obesity-related insulin resistance [15]. These effects of insulin resistance were blunted in PPARα null mice and recapitulated in MHC-PPARα mice, suggesting that PPARα is involved in mitochondrial biogenesis in the myocardium in the context of insulin resistance, which was previously not well-appreciated.

## 7. CONCLUSIONS

In summary, the heart requires a continuous and abundant source of substrate to meet it high-energy demands. In situations where energy needs change, such as heart failure, the heart must adapt and will utilize the most efficient source of substrate (glucose) to meet its needs. Similarly, when glucose availability becomes limited, as it does in fasting or diabetes, the heart will adapt and use fatty acid to meet its ATP requirements. PPARα and PGC-1α play a central role in this metabolic flexibility by driving robust changes in gene expression of key components of mitochondrial biogenesis and metabolism. However, it is still not entirely clear whether long-term PPARα-PGC-1α-mediated alterations in energy metabolism are adaptive versus maladaptive changes for both heart failure and diabetic cardiomyopathy.
